# Exploring the Use of Customized Links to Improve Electronic Engagement With Sexual and Reproductive Health Care Among Young African American Male Individuals: Web-Based Survey Study

**DOI:** 10.2196/48371

**Published:** 2024-04-24

**Authors:** Sandy Arena, Mackenzie Adams, Jade Burns

**Affiliations:** 1 School of Nursing University of Michigan Ann Arbor, MI United States

**Keywords:** African American, engagement, men’s health, recruit, recruitment, reproductive health, sexual behavior, sexual health behavior, sexual health, sexual transmission, sexually transmitted, social media, STIs, young adult, young adults

## Abstract

**Background:**

Research has shown that heterosexual African American male individuals aged 18-24 years have a higher prevalence of sexually transmitted infections (STIs) and are more likely to engage in risky sexual behavior. There is a critical need to promote sexual reproductive health (SRH) services among this population, especially in urban settings. Young African American male individuals use social media platforms to access health information, showcasing the potential of social media and web-based links as tools to leverage electronic engagement with this population to promote SRH care.

**Objective:**

This study aims to explore electronic engagement with young African American male individuals in discussions about SRH care. This paper focuses on the recruitment and social media marketing methods used to recruit young, heterosexual African American male individuals aged 18-24 years for the Stay Safe Project, a larger study that aims to promote SRH services among this population in Detroit, Michigan. We investigate the use of TinyURL, a URL shortener and customized tool, and culturally informed social media marketing strategies to promote electronic engagement within this population.

**Methods:**

Participants were recruited between December 2021 and February 2022 through various modes, including email listserves, Mailchimp, the UMHealthResearch website, X (formerly Twitter), Facebook, and Instagram. Images and vector graphics of African American male individuals were used to create social media advertisements that directed participants to click on a TinyURL that led to a recruitment survey for the study.

**Results:**

TinyURL metrics were used to monitor demographic and user data, analyzing the top countries, browsers, operating systems, and devices of individuals who engaged with the customized TinyURL links and the total human and unique clicks from various social media platforms. Mailchimp was the most successful platform for electronic engagement with human and unique clicks on the custom TinyURL link, followed by Instagram and Facebook. In contrast, X, traditional email, and research recruiting websites had the least engagement among our population. Success was determined based on the type of user and follower for each platform, whether gained in the community through sign-ups or promoted at peak user time and embedded and spotlighted on nontraditional media (eg, social media sites, blogs, and podcasts) for the user. Low engagement (eg, traditional email) from the target population, limited visibility, and fewer followers contributed to decreased engagement.

**Conclusions:**

This study provides insight into leveraging customized, shortened URLs, TinyURL metrics, and social media platforms to improve electronic engagement with young African American male individuals seeking information and resources about SRH care. The results of this study have been used to develop a pilot intervention for this population that will contribute to strategies for encouraging sexual well-being, clinic use, and appropriate linkage to SRH care services among young, heterosexual African American male individuals.

## Introduction

Nearly half of the 26 million people who are diagnosed with sexually transmitted infections (STIs) in the United States are younger than 25 years old [1]. Within this population, heterosexual African American male individuals aged between 18 and 24 engage in higher rates of risky sexual behavior (eg, early sexual initiation, inconsistent condom use, sex while under the influence, and multiple partners) and face a disproportionate burden of STIs compared to other adolescents and young adults [2-4].

In urban areas, this problem is particularly acute. African American male individuals who engage in low-risk sexual behavior, such as using condoms consistently or having sex with only 1 partner, are still at an increased risk of STIs due to risky social and sexual networks within urban communities that have high rates of violence, incarceration, and poverty [5-7]. A study by Marcell et al [8] found that among an urban sample of 70 African American and Hispanic young male individuals aged between 15 and 24 years, 67% reported having visited a health care provider in the past year, yet only 50% reported receiving HIV testing and only 39% reported receiving STI testing. These daunting statistics regarding sexual health indicate that young African American male individuals have a substantially greater need for sexual reproductive health (SRH) services (eg, preventative services, vaccination, diagnostic testing, and treatment) than other groups [9].

Studies show the significant factors in a young African American male individual’s decision to seek SRH services include concerns about confidentiality, self-confidence, and choice of provider, in addition to stigma, cost of care and contraceptives, and long wait times [8-10]. To explore the central phenomenon of SRH use and clinic access among young African American male individuals, community engagement with this population is crucial. Community engagement is particularly important for reducing HIV and STI disparities among young African American male individuals, and a lack of engagement with SRH care among this population can increase the risk of STIs and other health disparities [11]. Burns et al [12] found that social media can be used to effectively engage with young African American male individuals and provide them with information and resources regarding SRH care. Therefore, engaging with young African American male individuals electronically through social media may be a helpful strategy to ensure that they have access to the information and resources needed to make informed decisions about their SRH.

Social media platforms, particularly among African American adolescents and young adults, are already being used to seek general and sensitive health information due to their accessibility and anonymity [13]. Teadt et al [13] highlight the potential of social media platforms to serve as valuable tools for promoting sexual health awareness, prevention, and intervention development aimed at this population. For instance, in 2018, researchers from the Tulane School of Public Health and Tropical Medicine launched “Check It Nola” [14], a website for their research study geared toward using novel methods in promoting SRH care among heterosexual African American male individuals aged between 15 and 24 years in New Orleans, Louisiana. The “Check It” program used culturally informed marketing strategies (eg, using images of African American male individuals wearing crowns and the tagline “a king knows his status”) to resonate with young African American male individuals on popular social media platforms such as Instagram (Meta Platforms), X (formerly Twitter; X Corp), and Facebook (Meta Platforms), in addition to a website, as a means to engage them in SRH care [15]. This study seeks to create novel strategies similar to the “Check It” program by further exploring electronic engagement with SRH and preventative care services by young African American male individuals.

Following these existing approaches, we developed this study to explore an innovative approach to engaging with young African American male individuals about SRH care through culturally informed advertising on social media platforms (Instagram, Facebook, and X) in addition to traditional methods such as email, Mailchimp, and a participant recruiting website (UMHealthResearch). We used the TinyURL web service to create customized, shortened URLs that allowed us to track which social media and web-based platforms young African American male individuals were engaging with most. This study is part of the Stay Safe Project, a larger study which used a mixed method approach of surveying and focus groups to identify the positive and negative experiences of young African American male individuals when accessing SRH services at community health centers in a large city, the results of which will be reported elsewhere. It is important to recognize that community engagement is essential in order to reduce sexual health disparities among young African American male individuals. The use of social media and web-based links to promote SRH services and increase awareness may be used as an effective way to reach this population and be used to mediate health care–seeking behavior. This study describes the process for electronic engagement with young African American male individuals with regard to SRH services and may be used to promote long-term risk-reduction methods among young male individuals in the community.

## Methods

### Overview

This study examines electronic recruitment methods for young, heterosexual African American male individuals as part of a larger project, the Stay Safe Project, that seeks to promote SRH services for this population.

### Study Population

The priority population for this study was young, heterosexual African American male individuals. Inclusion criteria for the Stay Safe Project were: (1) aged between 18 and 24 years; (2) self-identify as male; (3) live in Detroit, Michigan; (4) self-identify as African American or Black; (5) self-identify as heterosexual and report having sex only with women in the last year; and (6) speak, read, and write in English.

### Recruitment

Participants were recruited between December 2021 and February 2022 through traditional email listserves, Mailchimp (an email marketing service or newsletter; Intuit), the UMHealthResearch website for web-based recruitment [16], X, and through Facebook and Instagram using paid advertising.

For each recruitment platform, we created a unique, shortened TinyURL link that led to a web-based recruitment survey for the study so we could track electronic engagement and user data. URL, which stands for Uniform Resource Locator, was first defined in 1994 as “the syntax and semantics for a compact string representation of a resource available through the internet” [17]. In modern terms, a URL is a unique address that identifies a specific web page on the internet. TinyURL is a URL shortening service that allows customers to create branded, customized URLs and track analytics for those URLs [18]. TinyURL allows customers to create shortened, customized links. Today, the terms “URL” and “link” are often used interchangeably. However, a link is defined as a clickable element on a web page that will lead to a specific URL [19]. Each TinyURL link was customized to the platform. All customized links directed participants to the same URL for our recruitment survey.

We offered a US $20 gift card to those who completed the survey, met the study criteria, and participated in the focus groups to incentivize participants to click on the links to our recruitment survey.

### Social Media Marketing and Analysis

Photo and banner advertisements were designed to capture the attention of young African American male individuals with the aim of recruiting eligible individuals for this study. Advertisements included images or vector graphics of African American male individuals with headlines calling for the participation of young African American male individuals and visible information on requirements and details about the focus group study, in addition to the US $20 gift card compensation. Advertisements were designed to grab the attention of social media users and were created with bright, appealing colors. The customized TinyURL links, which participants were directed to through our social media advertisements, helped the research team track total clicks, total unique clicks, and total human clicks on our survey link for each recruitment platform (Instagram, Facebook, X, traditional email, Mailchimp, and UMHealthResearch). TinyURL also allowed the research team to track world traffic (where in the world people were clicking on our link), the top browsers and devices people were using, and the popular days and times people were accessing our recruitment survey through Qualtrics (Silver Lake) from each custom link. The survey was developed to evaluate demographic data to determine eligibility for the study in addition to assessing satisfaction with SRH care and behavioral health risk characteristics (eg, sexual activity and substance use).

Additionally, we used Facebook and Instagram Insights (within Meta Business Suite) to further monitor and evaluate the performance of the recruitment campaigns, as well as the audience’s engagement with them. Insights is a tool offered by Meta that provides data and metrics about the performance of profile pages and individual posts on Facebook and Instagram. Using Insights, we were able to track content interactions such as the number of views, likes, comments, and shares, in addition to impressions, reach, link clicks, and profile visits on Facebook and Instagram. We were able to tailor our paid advertisements by sex, age range, and location (male individuals aged between 18 and 24 in Detroit). We tracked the times and days people were the most active on Facebook and Instagram and tailored our posting schedule accordingly with the hopes of increasing engagement with young African American male individuals in our community. Table 1 shows the definitions of terms we used for tracking electronic engagement.

**Table 1:** Terms and definitions.

Terms	Definitions
Total clicks	The total number of times a link was clicked on [20].
Unique clicks	The first click of a link by a unique user [20].
Human clicks	The total number of clicks on a link identified as authentic (from humans, as opposed to bots) [20].
World traffic	The countries and subregions where people were clicking on a link [20].
Top browser	The top browser used when a user clicks on a link to access and view websites (eg, Google Chrome [Google LLC], Safari [Apple Inc], Firefox [Mozilla], and Microsoft Edge [Microsoft Corporation]) [20].
Top operating system	The top operating system on a device used to click a link (eg, Microsoft Windows, macOS, iOS, and Android) [20].
Top device	The top device used to click on a link (eg, smartphones, computers, and tablets) [20].
Insights	Social media performance metric tool available for business accounts on Instagram and Facebook that shows the number of views, likes, comments, shares, impressions, reach, profile visits, and link clicks [21,22].
Impressions	The total number of times a post or ad has been seen. This includes both paid and unpaid impressions and may include multiple views by the same person [21,22].
Reach	The number of unique people that have seen your post or ad on a screen. This means that if an ad is seen by a person multiple times, it will only count as one impression [21,22].
Profile visits	The number of times a user has clicked on a post or ad to visit our Instagram or Facebook page [21,22].

### Ethical Considerations

The University of Michigan Institutional Review Board approved this study (HUM#00156338). All participants were provided with detailed information about the study before participating and were asked to provide their electronic informed consent before beginning the survey. Participants were assured their information would remain confidential and be stored on a secure, password-protected cloud service at the University of Michigan (U-M Dropbox).

## Results

### Overview

Overall, using TinyURL metrics allowed us to track important demographic and user information on individuals who clicked on our customized TinyURL links. This information, which is displayed in Table 2, includes the top countries, browsers, operating systems, and devices of the individuals who engaged with our customized TinyURL links. This information was collected in order to gain insight into electronic engagement with our customized TinyURL links and measure the success of our web-based marketing campaigns in reaching our target population. We have chosen to report both total human clicks and total unique clicks on our customized TinyURL links. When measuring successful electronic engagement, we have chosen to focus on total human clicks, as it provides a comprehensive overview of the activity generated by our links and represents whether the same individual clicked on a link more than once. Table 2 reports demographic and electronic user information based on total human clicks, as captured by the TinyURL platform. However, in our analysis we also discuss unique clicks since this information is measured only once, the first time that an individual clicks on the link.

Mailchimp was found to be our most successful platform for electronic engagement, with 245 human clicks and 106 unique clicks on the custom TinyURL link from December 2021 to March 2022. The most popular day users clicked on the TinyURL link for Mailchimp was Thursday, and the most popular times for users were morning and afternoon, occurring between 8 AM and 4 PM. The second-most successful platform was Instagram, which received 190 human clicks and 158 unique clicks from December 2021 to March 2022. The most popular day users clicked on the TinyURL link for Instagram was Sunday, with the clicks being evenly distributed throughout the day. The custom TinyURL link for Facebook found moderate success, receiving 56 human clicks and 46 unique clicks from December 2021 to March 2022. The most popular day users clicked on the custom TinyURL link for Facebook was Monday. Clicks for this link were evenly distributed throughout the day. The custom TinyURL link for X received 39 human clicks and 28 unique clicks from December 2021 to March 2022. Days and times users clicked on the TinyURL link for X were evenly distributed throughout the week and the day. The TinyURL link for traditional email received 31 human clicks and 24 unique clicks from December 2021 to March 2022. These clicks were evenly distributed throughout the week and throughout the day. The custom TinyURL link for UMHealthResearch received 15 human clicks and 10 unique clicks from December 2021 to March 2022. These clicks were evenly distributed throughout the week, falling mostly during weekdays, with the most popular time being in the afternoon and night, occurring between 12 PM and 12 AM.

**Table 2:** TinyURL metrics for Instagram, Facebook, X, traditional email, Mailchimp, and the UMHealthResearch website.

Metrics and ranks		Instagram			Facebook			X			Traditional email			Mailchimp			UMHealthResearch			
		Value	Clicks, n (%)	Value		Clicks, n (%)	Value		Clicks, n (%)	Value		Clicks, n (%)	Value		Clicks, n (%)	Value		Clicks, n (%)		
**Top countries**																				
	1	United States	96/190 (50.5)	United States		29/56 (50)	United States		21/34 (61.8)	United States		15/31 (48.4)	United States		105/224 (46.9)	United States		12/15 (80)		
	2	Ireland	38/190 (20)	Ireland		16/56 (28.6)	Germany		3/34 (8.8)	Kenya		2/31 (6.5)	Great Britain		55/224 (24.6)	—^a^		—		
	3	Kenya	28/190 (14.7)	Kenya		4/56 (7.1)	France		1/34 (2.9)	Canada		2/31 (6.5)	France		1/224 (0.5)	—		—		
**Top browsers**																				
	1	Chrome	109/188 (58)	Chrome		22/56 (43)	Chrome		12/34 (35.3)	Chrome		15/31 (48.4)	cURL^b^		92/224 (41.1)	cURL		6/15 (40)		
	2	Instagram	33/188 (17.6)	Facebook		11/56 (17.9)	cURL		10/34 (29.4)	cURL		4/31 (12.9)	Microsoft Edge		80/224 (35.7)	Internet Explorer		6/15 (40)		
	3	Facebook	20/188 (10.6)	Safari		7/56 (12.5)	Internet Explorer		7/34 (20.6)	Internet Explorer		4/31 (12.9)	Chrome		24/224 (10.7)	Chrome		2/15 (13.3)		
**Top operating systems**																				
	1	Android	100/184 (54.4)	Android		27/56 (55.8)	Windows		11/24 (45.8)	Android		16/25 (64)	Windows		120/128 (93.8)	Windows		6/9 (66.7)		
	2	iOS	38/184 (20.7)	iOS		11/56 (22)	Mac		5/24 (20.8)	Windows		8/25 (32)	Mac		5/128 (3.9)	Android		2/9 (22.2)		
	3	Windows	33/184 (18)	Windows		11/56 (22)	Android and Linux (tied)		3/34 (12.5)	Mac		1/25 (4)	iOS		3/128 (2.3)	Mac		1/9 (11.1)		
**Top devices**																				
	1	Smartphone	88/184 (47.8)	Smartphone		27/50 (54)	Desktop		20/24 (83.3)	Smartphone		16/25 (64)	Desktop		125/128 (97.7)	Desktop		7/9 (77.8)		
	2	Tablet	50/184 (27.2)	Desktop		12/50 (24)	Tablet		3/24 (12.5)	Desktop		9/25 (36)	Smartphone		3/128 (2.3)	Smartphone		2/9 (22.2)		
	3	Desktop	46/184 (25)	Tablet		11/50 (22)	Smartphone		1/24 (4.2)	—		—	—		—	—		—		

^a^Not available.

^b^cURL: client for URL.

### Instagram and Facebook Insights

In addition to TinyURL metrics, we also used Instagram Insights, Meta Business Suite, and Facebook Insights to measure the performance of our paid advertisements on the 2 platforms. The Instagram Insights feature for businesses allowed us to measure impressions, reach, profile visits, and link clicks for the paid advertisements that we posted on the platform. Using Instagram Insights, we measured impressions, reach, profile visits, and link clicks for 5 paid advertisements from December 2021 to February 2022 (Table 3). Additionally, the use of Meta Business Suite and Facebook Insights allowed us to measure impressions, reach, and link clicks for the posts that we paid to be boosted on the platform. Using Meta Business Suite and Facebook Insights, we measured impressions, reach, and link clicks for 2 paid advertisements in February 2022 (Table 4).

**Table 3:** Instagram Insights metrics.

Dates	US $/day	Impressions	Reach	Profile visits	Link clicks
December 18, 2021, to December 22, 2021	2	1437	738	4	2
January 27, 2022, to January 30, 2022	3	1850	1157	3	3
January 30, 2022, to February 2, 2022	3	1868	1214	6	9
February 10, 2022, to February 13, 2022	3	1355	1018	9	2
February 16, 2022, to February 18, 2022	4	1290	936	9	10

**Table 4:** Meta Business Suite and Facebook Insights metrics from paid advertisements.

Dates	US $/day	Impressions	Reach	Link clicks
February 10, 2022, to February 13, 2022	5	2001	1485	11
February 16, 2022, to February 18, 2022	5	2097	1427	9

### Survey Eligibility Results

The survey yielded 525 responses, of which 489 finished the survey and 32 were eligible for the study for focus groups. The survey respondents were removed from the eligible sample if they did not identify as African American or Black (16 removed), were outside of the age range of between 18 and 24 years old (27 removed), did not identify as heterosexual (23 removed), or did not reside in the Southeast Michigan area (391 removed). These exclusions were made to identify an eligible sample.

## Discussion

### Overview

Our findings suggest that Mailchimp was the most popular platform for electronic engagement, likely due to the fact that we had a well-curated list of email addresses from people who have previously engaged with our research, including colleagues, students, social media site followers, and community members (ie, individuals residing in Detroit, Michigan, or Southeastern Michigan). The use of Mailchimp allowed us to directly reach this audience and provide them with relevant and targeted content about our SRH care campaign and access to the customized link, resulting in high community engagement. In addition to Mailchimp, we also found that Instagram and Facebook were popular social media platforms for engaging with young people. Our results coincide with the fact that Instagram is reported to be an especially popular social media platform among young adults and teens, and Facebook remains a widely used platform by a majority of Americans [23].

On the other hand, we found X, traditional email, and UMHealthResearch to be our least successful platforms for electronic engagement. This may have been attributed to several reasons. First, our following on X was smaller than on our other platforms. Second, for emails that were sent, messages may have been filtered as spam and never opened. Traditional emails can be tracked after being sent but only after being opened if connected to an email tracking software system such as Mailchimp [24]. Finally, our advertisements on UMHealthResearch may have faced challenges in attracting engagement due to participants being required to create an account and undergo verification, resulting in a limited sample. UMHealthResearch promoted this study to only individuals who were already using the third-party recruitment platform, so the study advertisement was not as visible compared to other platforms.

The use of customized TinyURL links helped us track which social media platforms were the most effective in reaching young African American male individuals regarding SRH care. TinyURL allowed us to track clicks on each custom URL we created and see information such as country, browser, device, etc from each person. This information can be used to tailor future social media marketing campaigns about SRH to these platforms, which can help to ensure that young African American male individuals have access to the information and resources they need to make informed decisions about their sexual health. Additionally, customized TinyURL links are easy to share and remember, which can lead to stronger engagement, especially with younger populations. Overall, custom TinyURL links allowed us to track which specific posts and advertisements were the most engaging and which platforms were most effective in reaching people. The information found through tracking TinyURL metrics can then be used to create more effective and engaging content about SRH for young African American male individuals. In addition to informing strategic marketing and content creation, the information from TinyURL metrics can be used to engage young African American male individuals in events and research about SRH care advertised on social media.

By using culturally informed social media advertisements and customized TinyURL links, we were able to effectively engage with young African American male individuals, provide them with information and resources regarding SRH care, and encourage them to seek health care. The results of this study may be useful for improving the effectiveness of future social media marketing campaigns using culturally informed advertising to engage young African American male individuals in SRH care.

### Implications

Our findings suggest that customized TinyURL links can be an additional way to promote long-term risk-reduction methods among young male individuals in the community. Creating a TinyURL link for each platform we used to post content about SRH care allowed us to provide young African American male individuals with an easy way to access information and resources about SRH. By using customized TinyURL links, researchers can engage with young African American male individuals in a memorable, convenient, and trackable way on social media to help this population make informed decisions about their SRH. Custom TinyURL links are convenient and memorable enough to be easily shared between young male individuals, in person or using technology. Researchers using TinyURL can gain insight on how young African American male individuals engage with content and resources provided through those links. Additionally, by tracking which social media platforms are most effective in reaching young male individuals, researchers can target future social media marketing campaigns to these platforms. For example, a clinic seeking to engage with young African American male individuals in their community could use TinyURL to create custom links for their different social media platforms and track which links generate the most engagement. Using customized TinyURL links could inform the clinic’s social media marketing strategy on how to best engage with young African American male individuals about ongoing SRH care, from anonymous surveys to making an annual appointment to chatting with a health care provider virtually.

### Limitations

One limitation of this study was the unexpected engagement from users outside the United States, particularly in countries such as Ireland and Kenya, clicking on our links. In the case of Instagram and Facebook, despite us setting target audience parameters for our paid advertisements, such as location, age, and sex, we found that the platforms could not always accurately control who sees the advertisements and limit it to the demographic characteristics of our target population. Factors such as IP address blockers or users intentionally altering their location could also result in engagement from unexpected locations. Additionally, we promoted a US $20 gift card for eligible participants who completed the survey, which could have attracted users from different countries to click on our links, potentially resulting in engagement from a wider audience than initially intended. Further research would be needed to draw definitive conclusions about the reasons for engagement with specific countries. As a result, the generalizability of our findings may be limited.

Another limitation of this study was the high number of survey responses that were ineligible for the study. Out of 525 total responses, only 489 participants completed the survey, and only 32 were ultimately eligible for the study to participate in focus groups. This may be due to web-based social media targeting not being geographically limited enough or the focus on the city of Detroit not being prominent enough in the advertisements, leading to responses from participants who did not meet the study criteria. Additionally, social media platforms such as Instagram and Facebook, where we ran paid advertisements, did not offer the option to specify ethnicity or race when targeting an audience for advertisements, which may have resulted in a large number of responses from participants who did not align with our target population. Meta, the parent company of Facebook and Instagram, removed certain advertisement targeting options that included race, religion, sexual orientation, and political affiliation in January 2022 [25]. Meta’s detailed targeting advertising options are often changing and being refined, and options to target certain specific groups may not always be available [26]. Additionally, there is no guarantee that your advertisement will only be shown within your specific geographic target area; this poses an inherent challenge when using a recruitment strategy for your target population that is centered on social media [27]. This limitation makes it difficult to tailor our recruitment methods, making it difficult to ensure we reach the correct pool of participants. This makes social media advertising somewhat unpredictable, and the effectiveness of this method may be limited by this factor. This inherent limitation calls for a more tailored approach in the design and use of participant recruiting social media advertisements, in addition to rigorous screening to ensure the internal validity of the results.

In reviewing TinyURL metrics for our links, there were missing data as we noticed that TinyURL could not capture all information on country, browser, operating system, and device for all recorded total human clicks for the links we created. For example, with Instagram, while TinyURL had data regarding the top countries for all 190 total human clicks when it came to reviewing the top browsers, TinyURL only had information for 188 of those clicks. For top operating systems, TinyURL only had information for 184 of those clicks. TinyURL identifies these data using user agent strings [20], which may be blocked by specific devices or browsers due to privacy settings. This aspect is important as it limits the collection of these data from users who click on customized links. However, this limitation may have had minimal impact on the insights and conclusions, as information was still viewable to the majority of the users who were able to access and click our links.

In addition, our use of social media as a recruitment strategy may have limited the pool of participants for this study. By using this method, we may have inadvertently excluded those without internet access and those who do not use or do not have social media accounts. It is important to note that social media approaches typically come with this selection bias, and it is important to acknowledge that this may have recruited from a skewed pool of applicants.

Furthermore, we encountered issues with spam responses and participants who may have been solely interested in winning the gift card, which could have affected the validity of our results. A solution to this would be setting up multiple captchas at different phases of the survey (eg, eligibility or payment) or advertisement to assess for human use.

### Conclusion

Using customized, shortened URLs and TinyURL metrics to study social media engagement specifically to promote SRH care among young African American male individuals is innovative and provides future researchers with the potential to discover valuable insights into how to reach this population both locally and globally with sexual health information and resources. Social media is a powerful tool that can be leveraged to reach young people with information about SRH care. TinyURL links offer a modern, quick means to engage with people about SRH care and track their electronic engagement. Customized TinyURL links, in conjunction with culturally informed advertisements and content, can be shared on popular social media platforms to engage with young African American male individuals seeking information and resources about SRH care. The findings from this study have the potential to inform future literature on the topic of improving electronic engagement and content creation with SRH care in unique spaces among young African American male individuals.

## Abbreviations

SRH: sexual and reproductive health
STI: sexually transmitted infection

## Data Availability

The data sets generated and analyzed during this study are not publicly available due to ethical concerns about maintaining the confidentiality of survey participants’ personal information but are available from the corresponding author on reasonable request.
